# Effectiveness of Serious Games for Improving Executive Functions Among Older Adults With Cognitive Impairment: Systematic Review and Meta-analysis

**DOI:** 10.2196/36123

**Published:** 2022-07-25

**Authors:** Alaa Abd-alrazaq, Dari Alhuwail, Arfan Ahmed, Mowafa Househ

**Affiliations:** 1 AI Center for Precision Health Weill Cornell Medicine-Qatar Doha Qatar; 2 Division of Information and Computing Technology, College of Science and Engineering Hamad Bin Khalifa University Qatar Foundation Doha Qatar; 3 Information Science Department College of Life Sciences Kuwait University Kuwait Kuwait; 4 Health Informatics Unit Dasman Diabetes Institute Kuwait Kuwait

**Keywords:** serious games, cognitive training, exergames, executive functions, mild cognitive impairment, Alzheimer disease, dementia, systematic reviews, meta-analysis

## Abstract

**Background:**

Executive functions are one of the known cognitive abilities that decline with age. They are the high-order cognitive processes that enable an individual to concentrate, plan, and take action. Serious games, which are games developed for specific purposes other than entertainment, could play a positive role in improving executive functions. Several systematic reviews have pooled the evidence about the effectiveness of serious games in improving executive functions; however, they are limited by some weaknesses.

**Objective:**

This study aims to investigate the effectiveness of serious games for improving executive functions among older adults with cognitive impairment.

**Methods:**

A systematic review of randomized controlled trials (RCTs) was conducted. To retrieve relevant studies, 8 electronic databases were searched. Further, reference lists of the included studies and relevant reviews were screened, and we checked studies that cited our included studies. Two reviewers independently checked the eligibility of the studies, extracted data from the included studies, assessed the risk of bias, and appraised the quality of the evidence. We used a narrative and statistical approach, as appropriate, to synthesize results of the included studies.

**Results:**

Of 548 publications identified, 16 RCTs were eventually included in this review. Of the 16 studies, 14 studies were included in 6 meta-analyses. Our meta-analyses showed that serious games are as effective as no or passive interventions at improving executive functions (*P*=.29). Surprisingly, conventional exercises were more effective than serious games at improving executive functions (*P*=.03). Our subgroup analysis showed that both types of serious games (cognitive training games, *P*=.08; exergames, *P*=.16) are as effective as conventional exercises at improving executive functions. No difference was found between adaptive serious games and nonadaptive serious games for improving executive functions (*P*=.59).

**Conclusions:**

Serious games are not superior to no or passive interventions and conventional exercises at improving executive functions among older adults with cognitive impairment. However, our findings remain inconclusive due to the low quality of the evidence, the small sample size in most included studies, and the paucity of studies included in the meta-analyses. Accordingly, until more robust evidence is available, serious games should not be offered by health care providers nor used by patients for improving executive functions among older adults with cognitive impairment. Further reviews are needed to assess the long-term effect of serious games on specific executive functions or other cognitive abilities among people from different age groups with or without cognitive impairment.

**Trial Registration:**

PROSPERO CRD42021272757; https://www.crd.york.ac.uk/prospero/display_record.php?RecordID=272757

## Introduction

### Background

Globally, the older adult population is rapidly increasing at unprecedented rates. By the year 2050, approximately 2 billion people are expected to live to over 65 years old [1]. As people age and live longer, it is unclear if their additional years of living are enjoyed in good health [2]. Generally, as people grow older, their risk of experiencing cognitive impairment increases [3]. After the age of 70 years, older adults will, unfortunately, experience physical and mental multimorbidities [4]; they will require special care and attention because of the emergence of multiple progressive health complications, including declining mental and cognitive functions, noncommunicable diseases (eg, diabetes), vision impairments, hearing loss, and physical ailments [5]. Additionally, aging is often accompanied by various social problems, including economic or financial insecurity, isolation, and loneliness [6].

For older adults, the World Health Organization (WHO) estimates that nearly 7% of the total disability-adjusted life years (DALYs) are attributed to mental and neurological disorders [7]. Among the top culprits causing the progressive decline in cognitive functions and abilities is mild cognitive impairment (MCI), which in turn increases the risk for developing dementia and Alzheimer disease (AD) [8]. In the United States alone, it is estimated that, by 2050, approximately 13.8 million older adults will have AD-related dementia [9]. Economically, European countries estimated the toll of AD alone at €232 billion in 2015, and it is expected that this cost will double by 2040 [10]. The toll brought by the declining mental and cognitive functions of older adults places a large financial burden on public health. The stress brought by the declining mental and cognitive functions of older adults is further exacerbated by effects on the older adult’s family members, caregivers, and society. Therefore, the WHO recommended that the prevention of mental and cognitive decline is to be ranked as a global mental health priority [11].

One of the cognitive abilities that decline by age is executive function. Executive functions are essential for flexible, adaptive, and goal-oriented behavior [12]. Executive functions can be referred to as the high-order cognitive processes that enable an individual to concentrate, plan, and take actions [12]. Working memory, flexible thinking, and self-control are among the mental qualities that comprise executive function. Every day, we employ these abilities to learn, work, and govern our lives. Executive function issues can make it difficult to focus, follow directions, and manage emotions, to name a few things. Executive functions can generally be grouped into 3 core processes: (1) inhibiting predominant responses and controlling attention; (2) switching between tasks and cognitive flexibility; and (3) updating, retaining, and processing information [13,14]. Research suggests several ways to improve executive functions, including both pharmacological and nonpharmacological interventions.

With the explosive advances in technology, evidence suggests that computerized nonpharmacological interventions, including serious games, could play a positive role in improving executive functions [11]. Serious games are defined as games that are developed for specific purposes other than entertainment such as education, prevention, screening, diagnosing, and therapeutic rehabilitation [15,16]. Serious games had shown promising results in improving attention, concentration, and working memory [17]. Depending on their therapeutic modality, serious games may exist in a variety of formats, including (1) exergames, or videogames that require physical exercise as part of playing the game; (2) cognitive training games that aim to maintain or improve users’ cognitive abilities (eg, executive functions, memory, learning); (3) computerized cognitive behavioral therapy (CBT) games, which are video games that provide CBT for the users; and (4) biofeedback games, which are video games that utilize electrical sensors attached to the participant to receive information about the participant’s body state (eg, electrocardiogram sensors) and seek to influence some of the player’s body functions (eg, heart rate). With the increasing access and ubiquity of handheld computers and smart devices, serious games continue to become more abundant via videogame consoles, personal computers, and, more recently, smartphones and tablets [11].

### Research Gap and Aim

There are many studies that have examined the effectiveness of serious games in improving executive functions. Conducting systematic reviews to summarize the evidence in these studies is important to draw conclusions about the effectiveness of serious games in improving executive functions. Several systematic reviews have pooled findings of these studies. However, these reviews (1) focused on older adults without cognitive impairment [11,18-21], (2) included quasiexperiments or pilot randomized controlled trials (RCTs) [19,21-23], (3) did not assess the quality of the meta-analyzed evidence [11,19,22-24], (4) only focused on a specific type of serious game such as cognitive training games [11,20,22,24] and exergames [19,21,23], or (5) did not compare the effect of serious games with that of a specific comparator (eg, no intervention, conventional exercises, conventional cognitive activities) [11,19-24]. To address these gaps, the aim of this review was to investigate the effectiveness of serious games for improving executive functions among older adults with cognitive impairment.

## Methods

The Preferred Reporting Items for Systematic Reviews and Meta-Analyses (PRISMA) guidelines (Multimedia Appendix 1) [25] were followed to conduct this systematic review and meta-analysis. The protocol for this review is registered at the International Prospective Register of Systematic Reviews (PROSPERO; ID: CRD42021272757).

### Search Strategy

#### Search Sources

For the purpose of this review, the following 8 databases were searched: MEDLINE (via Ovid), PsycINFO (via Ovid), EMBASE (via Ovid), CINAHL (via EBSCO), IEEE Xplore, ACM Digital Library, Scopus, and Google Scholar. Searches were completed on November 10, 2021, by the first author, and an automatic alert was set up and ran its course for 8 weeks (ending on December 5, 2021). Only the first 10 pages (ie, 100 hits) in Google Scholar were considered because it returns a large number of studies that are automatically ordered based on their relevance [26]. We conducted backward reference list checking (ie, screening of reference lists of the included studies and relevant reviews). Finally, the studies that cited the included studies were screened (ie, forward reference list checking).

#### Search Terms

To develop the search query, we consulted 2 experts in digital mental health. The search terms included those related to the target population (eg, cognitive disorder), target intervention (eg, serious games), and target study design (eg, RCTs). Multimedia Appendix 2 summarizes the search query that was used to search each of the 8 databases.

### Study Eligibility Criteria

Only RCTs that evaluated the effectiveness of serious games for improving executive functions among older adults with cognitive impairment were included in this study. Serious games that were available on any digital platform, such as PCs, consoles (eg, Xbox, PlayStation), mobile phones, tablets, handheld devices, Nintendo, or any other computerized device, were included in this study. Furthermore, gaming had to be a key component of the intervention and used purely for therapeutic purposes. Studies combining serious games with other interventions were included if the control group received the same adjacent intervention. Nondigital games (eg, paper-and-pencil games or board games), as well as those used for monitoring, screening, diagnosis, and research, were excluded.

The population of interest was adults over 60 years old with any type of cognitive impairment or disorder (MCI, AD, or dementia). Their diagnosis had to be confirmed by checking the inclusion criteria or baseline scores against defined diagnostic criteria (eg, Mini-Mental State Examination [MMSE]). Studies about older adults without cognitive impairment, health care providers, and caregivers were excluded. No restrictions were applied regarding gender and ethnicity.

Regardless of the tool used to measure the outcome, the outcome of interest in this review was executive functions. This review did not focus on a specific executive function. Studies were excluded if they focused on only cost effectiveness, acceptance, feasibility, satisfaction, or cognitive abilities other than executive functions. This review focused on outcome data collected just after the intervention (postintervention data), rather than data collected later (follow-up data).

For practical reasons, only studies in the English language were eligible for inclusion. Although we considered all types of RCTs (parallel, cluster, crossover, and factorial) in this review, pilot RCTs, quasiexperiments, observational studies, and reviews were excluded. Research published in journals, conference proceedings, and dissertations from 2010 onwards were included. Those published as conference abstracts, conference posters, commentaries, proposals, and editorials were excluded. No restrictions related to the country of publication, comparator, and study settings were applied.

### Study Selection

The following steps were followed to identify relevant studies. First, the obtained studies were imported into EndNote to identify and delete duplicate items. Second, the titles and abstracts of all retrieved studies were evaluated by 2 reviewers working independently. Third, the 2 reviewers independently checked the entire texts of the studies included in the previous step. All disagreements were resolved via discussion between the reviewers. The interrater agreements (Cohen κ) in steps 2 and 3 were 0.86 and 0.94, respectively.

### Data Extraction

The 2 reviewers extracted data from included studies independently using Microsoft Excel. Before extracting data, we pilot tested the data extraction form with 2 of the included studies. Disagreements between the reviewers were settled through discussions between both reviewers. Multimedia Appendix 3 presents the data extraction form used to extract data from the included studies. First and corresponding authors were contacted in an attempt to retrieve metrics such as mean, standard deviation, and sample size if they were unavailable from the published studies.

### Risk of Bias Appraisal

The 2 reviewers used the Risk-of-Bias 2 (RoB-2) tool [27] to independently appraise the risk of bias in the included studies. The RoB-2 tool evaluates the risk of bias in 5 areas of RCTs: randomization process, deviations from intended interventions, missing outcome data, measurement of the outcome, and selection of the result [27]. All disagreements were resolved via discussion between the reviewers. The interrater agreement between the reviewers was 0.90.

### Data Synthesis

To summarize the collected data, narrative and statistical methods were used. Texts and tables were used to describe the characteristics of the included studies (demographic, intervention, comparison, and outcome measures) in our narrative synthesis. The results of the experiments were aggregated and classified by comparator: no or passive intervention control, conventional exercises, and other serious games. Meta-analyses were performed when 2 or more studies from the same comparator submitted sufficient data (ie, mean, standard deviation, and number of participants in each intervention group). Meta-analysis was performed using Review Manager (RevMan 5.4). The standardized mean difference (SMD; Cohen *d*) was used to estimate the overall effect of each study as the type of data for the outcome of interest (executive functions) was continuous and instruments used to evaluate the outcome were diverse among the included trials. We selected a random effects model for the analysis due to the excessive clinical heterogeneity among the meta-analyzed research in terms of serious game characteristics (eg, types, duration, frequency, and period), population characteristics (eg, sample size, mean age, and health condition), and outcome measures (ie, tools and follow-up period).

If there was a statistically significant difference between the groups when performing a meta-analysis, we sought to further investigate whether it was clinically significant. The term minimal clinically important difference (MCID) refers to the smallest change in measurement results that the patient considers reasonable and important enough to justify a change in treatment. The MCID boundaries were computed as ±0.5 times the SMD of the meta-analyzed studies.

To evaluate the degree and statistical significance of heterogeneity in the meta-analyzed studies, we calculated I^2^ and a chi-square *P* value, respectively. A chi-square *P* value ≤.05 suggests heterogeneous meta-analyzed studies [28]. The degree of heterogeneity was judged insignificant, moderate, substantial, or considerable when I^2^ ranged from 0% to 40%, 30% to 60%, 50% to 90%, or 75% to 100%, respectively [28].

To assess the overall quality of the evidence obtained from the meta-analysis, we used the Grading of Recommendations Assessment, Development and Evaluation (GRADE) approach [29]. The GRADE approach appraises the quality of evidence based on 5 domains: risk of bias, inconsistency (ie, heterogeneity), indirectness, imprecision, and publication bias [29]. The 2 reviewers independently evaluated the overall quality of the meta-analyzed evidence, and the differences in decisions were addressed via discussion. The interrater agreement between the reviewers was 0.89 [30].

## Results

### Search Results

**Figure 1:**
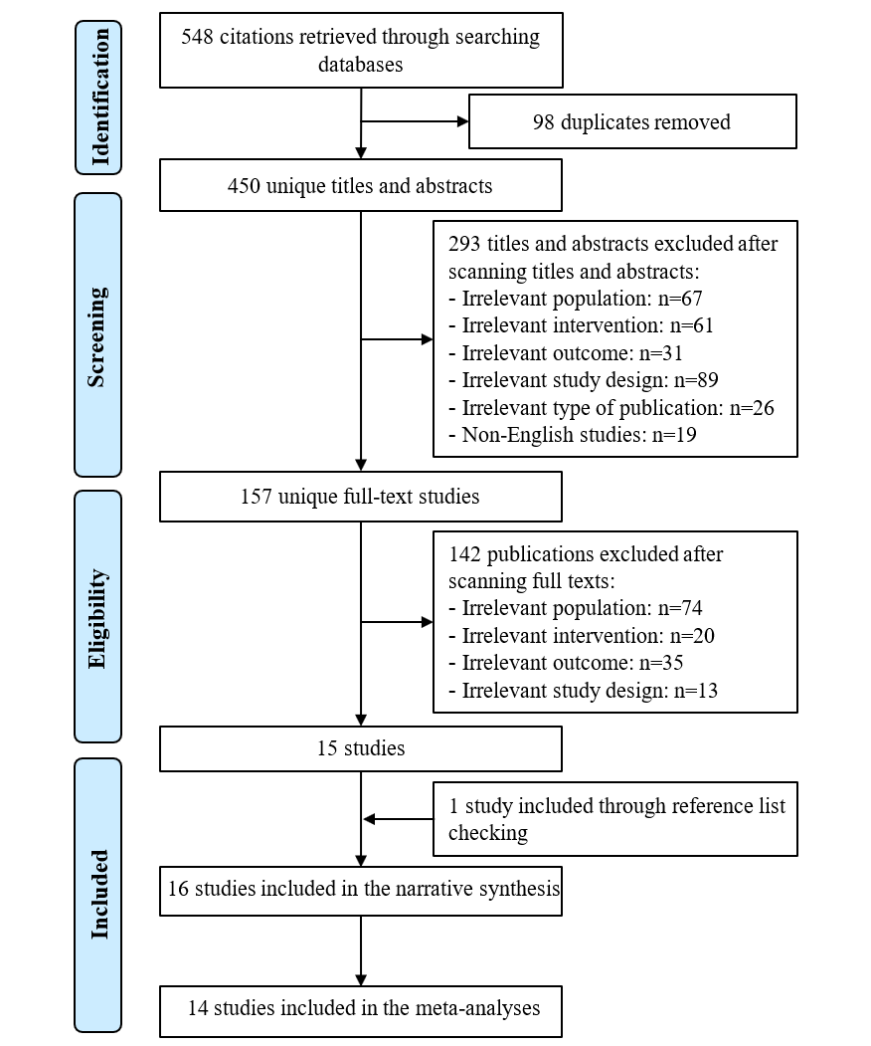
Flow chart of the study selection process.

By searching the 8 electronic databases, 548 records were retrieved (Figure 1). Of these records, 98 duplicates were excluded using the software EndNote. Checking titles and abstracts of the remaining records led to excluding 293 records for the following reasons: (1) Participants were younger than 60 years and/or without cognitive impairment (n=67); (2) interventions were not serious games (n=61); (3) the outcome was not executive functions (n=31); (4) study design was not an RCT (n=89); (5) studies were not peer-reviewed articles, theses, or conference proceedings (n=26); and (6) they were published in languages other than English (n=19). Reading the full text of the remaining 157 publications led to excluding 142 publications for the following reasons: (1) Participants were younger than 60 years and/or without cognitive impairment (n=74), (2) interventions were not serious games (n=20), (3) the outcome was not executive functions (n=35), and (4) study design was not an RCT (n=13). One additional study was found through backward reference list checking. In total, 16 RCTs were included in the current review [31-46]. All studies were included in meta-analyses except 2 studies [45,46].

### Characteristics of Included Reviews

The included studies were published between 2013 and 2021 (Table 1). The included studies originated in 11 different countries, with a roughly equal proportion of research in each country. There was a general equal distribution of studies in these countries. Except for 1 book chapter, all included papers were peer-reviewed academic publications. The trial type used in the most included studies was parallel RCTs (n=14).

**Table 1:** Characteristics of studies and population.

First author	Year	Country	Publication type	RCT^a^ type	Sample size	Mean age (years)	Sex (male), %	MMSE^b^ score	Health condition	Setting
Cavallo [31]	2016	Italy	Journal article	Parallel	80	76.4	36.3	22.9	AD^c^	Clinical
Finn [32]	2015	Australia	Journal article	Parallel	31	75.6	71	28.1	MCI^d^	Clinical
Yang [33]	2017	South Korea	Journal article	Parallel	20	71	70	23.1	AD	Clinical
Zhuang [34]	2013	China	Journal article	Parallel	33	83.1	24.2	10.2	MCI, dementia	Clinical
Thapa [35]	2020	South Korea	Journal article	Parallel	68	72.7	23.5	26.2	MCI	Clinical
Tarnanas [36]	2014	Greece	Book chapter	Parallel	114	70.3	39	26.4	MCI	Clinical
Singh [37]	2014	Australia	Journal article	Factorial	100	70.1	32	27	MCI	Community
Amjad [38]	2019	Pakistan	Journal article	Parallel	44	NR^e^	NR	24	MCI	Clinical
Hagovská [39]	2016	Slovakia	Journal article	Parallel	80	67	51.2	26.4	MCI	Clinical
van Santen [40]	2020	Netherlands	Journal article	Cluster	112	79	53.5	18.6	Dementia	Clinical
Karssemeijer [41]	2019	Netherlands	Journal article	Parallel	115	79.9	53.9	22.4	Dementia	Clinical & community
Liao [42]	2021	Taiwan	Journal article	Parallel	61	81.5	32.6	22.9	MCI	Community
Flak [43]	2019	Norway	Journal article	Parallel	85	66	66.7	NR	MCI	Clinical
Hyer [44]	2016	United States	Journal article	Parallel	68	75.2	47.1	26	MCI	Community
Park [45]	2017	South Korea	Journal article	Parallel	78	67.3	53.8	26.5	MCI	Community
Lee [46]	2018	South Korea	Journal article	Parallel	20	74.3	40	17.9	MCI, AD, dementia	Clinical

^a^RCT: randomized controlled trial.

**^b^**MMSE: Mini-Mental State Examination.

^c^AD: Alzheimer disease.

^d^MCI: mild cognitive impairment.

^e^NR: not reported.

The sample size in the included studies ranged from 20 to 115, with an average of 69.3. The average age of participants in the 15 studies was 74 years, with a range of 67 years to 83 years. The percentage of men reported in 15 studies ranged from 24.2% to 71%, with an average of 46.3%. Participants in the included studies had a mean MMSE score of 23.2, with a range of 10.2 to 28.1. Participants in the included studies had MCI (n=10), AD (n=2), dementia (n=2), MCI and dementia (n=1), and all 3 (MCI, AD, and dementia; n=1). Participants were recruited from clinical settings (n=11), the community (n=4), and clinical and community settings (n=1).

In 14 of the studies considered, serious games were employed alone as therapies, whereas the other 2 studies combined serious games with other interventions (Table 2). We identified 18 distinct serious games used in the studies; more than 1 game was used in certain studies. Serious games in the included trials were divided into 2 categories depending on the treatment modality they provide: cognitive training games (n=13) and exergames (n=3). In 14 studies, games were created with a “serious” objective from the start (designed serious games). Games in the remaining 2 studies, on the other hand, were not planned as serious games from the start but were instead used for a serious purpose (purpose-shifted games). Computers were the most popular platforms for playing games in the included studies (n=10). In most studies (n=11), serious games were played under the supervision of health care providers or carers. The game durations in the included studies ranged from 25 minutes to 100 minutes. The frequency of playing the games ranged from 2 times to 7 times per week, but it was 3 times per week in roughly one-third (6/16, 38%) of the studies. The duration of interventions ranged from 3 weeks to 25 weeks but was less than 13 weeks in the majority of studies (12/16, 75%).

The comparison groups in 7 studies received no or passive interventions (eg, reading newspaper articles, surfing the internet, watching a documentary program), whereas active interventions (eg, conventional exercises, other serious games) were conducted in 8 studies (Table 3). Two studies delivered both active as well as passive interventions as comparators. The duration of the active comparators ranged between 25 minutes and 100 minutes. The active comparators were used between once a week and 7 times a week. The duration of the active comparators ranged from 4 weeks to 25 weeks. The outcome of interest (ie, executive functions) was measured using 13 different tools, but the Trail Making Test B (TMT-B) was the most commonly used tool by the included studies (8/16, 50%). In all included studies, the outcome of interest was measured immediately after the intervention, and the longest follow-up period was 74 weeks. The number of participants who dropped out ranged from 0 to 28.

**Table 2:** Characteristics of interventions.

First author	Intervention	Serious game name	Serious game type	Serious game genre	Platform	Supervision	Duration (minutes)	Frequency (times/week)	Period (weeks)
Cavallo [31]	Serious games	Brainer	Cognitive training game	Designed	PC	Supervised	30	3	12
Finn [32]	Serious games	E-Prime	Cognitive training game	Designed	PC	Supervised	NR^a^	2	4
Yang [33]	Serious games	Brain-Care	Cognitive training game	Designed	PC	Unsupervised	60	2	12
Zhuang [34]	Serious games + sham exercises	NR	Cognitive training game	Designed	PC	Supervised	75	3	24
Thapa [35]	Serious games	Juice making, Crow Shooting, Love house, Fireworks	Cognitive training game	Designed	VR^b^ headset, hand controllers	Supervised	100	3	8
Tarnanas [36]	Serious games	Virtual Reality Museum	Cognitive training game	Designed	VR headset	Supervised	90	2	21
Singh [37]	Serious games	COGPACK	Cognitive training game	Designed	PC	Supervised	75	2	25
Amjad [38]	Serious games	Body and Brain Exercises	Cognitive training game	Purpose-shifted	Xbox console, Kinect	Supervised	25-30	5	6
Hagovská [39]	Serious games + conventional exercises	CogniPlus	Cognitive training game	Designed	PC	Supervised & unsupervised	30	2	10
van Santen [40]	Serious games	NR	Exergame	Designed	Stationary bike & screen	Unsupervised	NR	5	25
Karssemeijer [41]	Serious games	NR	Exergame	Purpose-shifted	Stationary bike & screen	Supervised	30-50	3	12
Liao [42]	Serious games	Tano and LongGood	Exergame	Designed	Kinect, VR headset	Supervised	60	3	12
Flak [43]	Serious games	Cogmed	Cognitive training game	Designed	PC	Unsupervised	30-40	5	5
Hyer [44]	Serious games	Cogmed	Cognitive training game	Designed	PC	Supervised & unsupervised	40	7	5-7
Park [45]	Serious games	CoTras	Cognitive training game	Designed	PC	Supervised	30	3	10
Lee [46]	Serious games	Bettercog	Cognitive training game	Designed	PC	Supervised	30	4	3

^a^NR: not reported.

^b^VR: virtual reality.

**Table 3:** Characteristics of comparators and outcomes.

First author	Comparator	Duration (minutes)	Frequency (times/week)	Period (weeks)	Outcome measures	Follow-up	Attrition, n
Cavallo [31]	Control	NA^a^	NA	NA	HSCT^b^, LVF^c^, BT^d^	Postintervention, 24-week follow-up	4
Finn [32]	Control	NA	2	4	D-KEFS^e^	Postintervention	7
Yang [33]	Control	NA	NA	NA	COWAT^f^	Postintervention	0
Zhuang [34]	Control	NA	NA	NA	ACE-R-F^g^	Postintervention	10
Thapa [35]	Control	30-50	1	8	TMT-B^h^	Postintervention	2
Tarnanas [36]	1: control; 2: conventional cognitive activities	90	2	21	TMT-B, LVF	Postintervention	9
Singh [37]	1: conventional exercises + sham cognitive training; 2: serious games + conventional exercises; 3: control	1: 75; 2: 100; 3: 60	2	25	COWAT, CF^i^, WAIS-III-S^j^, WAIS-III-M^k^	Postintervention, 74-week follow up	14
Amjad [38]	Conventional exercises	25-30	5	6	TMT-B	Postintervention	6
Hagovská [39]	Conventional exercises	30	7	10	ACE-WP^l^	Postintervention	2
van Santen [40]	Conventional exercises	NA	5	25	TMT-B	Midintervention, postintervention	28
Karssemeijer [41]	1: conventional exercises (aerobic exercises); 2: conventional exercises (relaxation and flexibility exercises)	30-50	3	12	TMT-B, LVF, RSCT^m^	Midintervention, postintervention, 24-week follow-up	23
Liao [42]	Conventional exercises	60	3	12	TMT-B, EXIT-25^n^	Postintervention	15
Flak [43]	Nonadaptive serious game	30-40	5	5	D-KEFS-CWIT3^o^, D-KEFS-CWIT4^p^, D-KEFS-VFTLF^q^, D-KEFS-VFTCF^r^, D-KEFS-VFTCS^s^	Postintervention, 4-week follow-up, 16-week follow-up	17
Hyer [44]	Nonadaptive serious game	40	7	5-7	TMT-B	Postintervention, 12-week follow-up	9
Park [45]	Serious game (exergames)	30	3	10	TMT-B	Postintervention	0
Lee [46]	Serious game (targeting attention and memory)	30	4	3	SNSB-II^t^	Postintervention	1

^a^NA: not applicable.

^b^HSCT: Hayling Sentence Completion Test.

^c^LVF: latter verbal fluency.

^d^BT: Brixton test.

^e^D-KEFS: Delis–Kaplan Executive Function System.

^f^COWAT: Controlled Oral Word Association Test.

^g^ACE-R-F: Addenbrooke’s Cognitive Examination-Revised-fluency.

^h^TMT-B: Trail Making Test B.

^i^CF: category fluency.

^j^WAIS-III-S: Wechsler Adult Intelligence Scale III-Similarities.

^k^WAIS-III-M: Wechsler Adult Intelligence Scale III-Matrices.

^l^ACE-WP: Addenbrooke's Cognitive Examination-Word production.

^m^RSCT: Rule Shift Cards Test.

^n^EXIT-25: Executive Interview 25.

^o^D-KEFS-CWIT3: Delis–Kaplan Executive Function System-Color Word Interference Test 3.

^p^D-KEFS-CWIT4: Delis–Kaplan Executive Function System-Color Word Interference Test 4.

^q^D-KEFS-VFTLF: Delis–Kaplan Executive Function System-Verbal Fluency Test Letter Fluency.

^r^D-KEFS-VFTCF: Delis–Kaplan Executive Function System-Verbal Fluency Test Category Fluency.

^s^D-KEFS-VFTCS: Delis–Kaplan Executive Function System-Verbal Fluency Test Category Switching.

^t^SNSB-II: Seoul Neuropsychological Screening Battery 2nd edition.

### Results of Risk of Bias Appraisal

**Figure 2:**
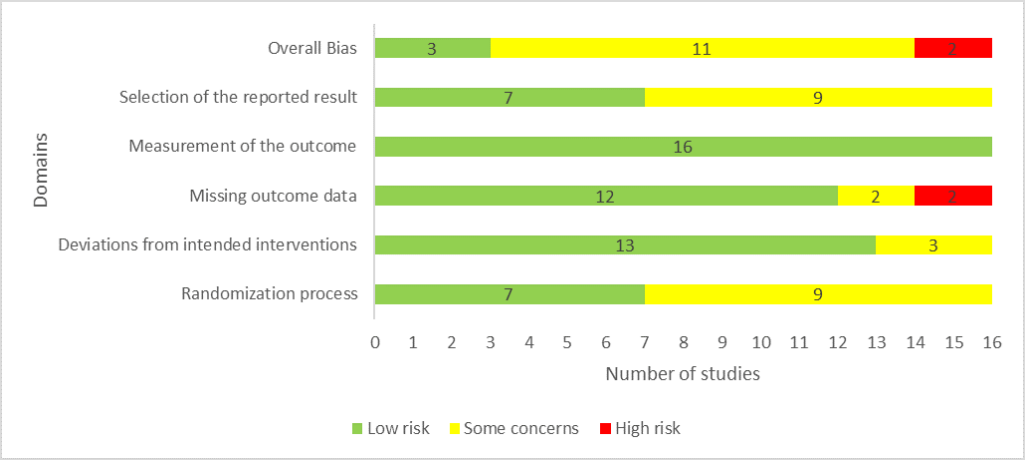
Review authors’ judgments about each "Risk of bias" domain.

In 10 studies, participants were randomly assigned to groups using a proper random allocation sequence. Half of the included studies concealed the allocation sequence until participants were assigned to interventions. Groups were comparable at baseline in 15 studies. Accordingly, 7 of the 16 studies were judged to have a low risk of bias in the “randomization process” domain (Figure 2).

Participants were aware of the assigned interventions during the trial in 12 studies. In 14 studies, individuals who delivered the interventions to the participants were aware of the assigned interventions. There was no evidence that the experimental contexts led to a deviation from the intended intervention in 14 studies. All included studies except for 1 study estimated the effect of the intervention using appropriate analysis methods (eg, intention-to-treat analysis). Consequently, 13 of the 16 studies were judged to have a low risk of bias in the “deviations from the intended interventions” domain (Figure 2).

In 6 studies, outcome data were available for more than 95% of the participants. In only 1 study, there was evidence that the findings were not biased by missing outcome data. The missing outcome data could be related to participants’ health status in 3 studies. According to these judgments, the risk of bias due to missing outcome data was low in 12 studies (Figure 2).

In all included studies, executive function was examined using appropriate measures, and measurement methods were comparable across intervention groups. In 6 studies, the assessor of the outcome was aware of the assigned interventions. In all studies, assessment of the outcome may not have been affected by knowledge of the intervention received. Therefore, the risk of bias in the “measuring the outcome” domain was rated as low in all studies (Figure 2).

Of the studies, 7 published their protocol in sufficient detail. In all studies, reported outcome measurements did not differ from those specified in the analysis plan, and there is no evidence that studies selected their results from many results produced from multiple eligible analyses of the data. Based on these judgments, 7 studies were judged to have a low risk of bias in the “selection of the reported results” domain (Figure 2).

In the last domain, “overall bias,” 3 studies were judged to be at low risk of bias given that it was rated to be at low risk of bias for all other domains. Because they had some issues in at least one of the domains and were not at high risk for any domain, 11 studies raised some concerns in the domain of overall bias. The risk of bias was rated high in 2 studies, as they were judged as having a high risk of bias in at least one domain. Reviewers’ judgments about each “risk of bias” domain for each included study are presented in Multimedia Appendix 4.

### Results of Studies

#### Serious Games Versus No or Passive Interventions

**Figure 3:**
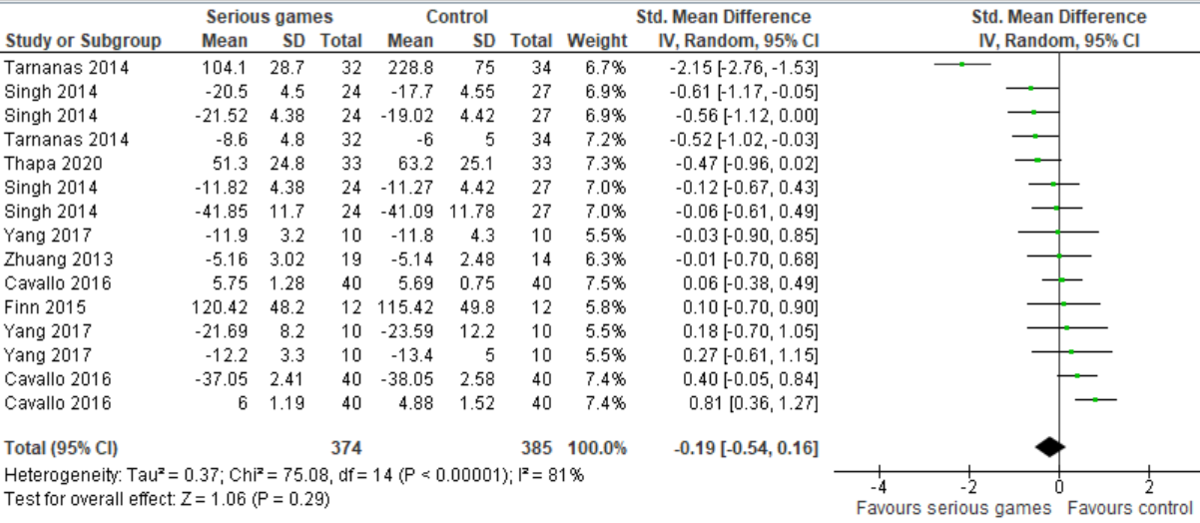
Forest plot of 7 studies (15 comparisons) comparing the effect of serious games to control on executive functions.

In 7 studies [31-37], the effect of serious games was compared with a control (no or passive intervention). Passive interventions refer to interventions that do not have a known effect on the measured outcome such as reading newspaper articles, surfing the internet, and watching a documentary program. Of these studies, 4 assessed executive function using more than one measure [31-33,36]. Therefore, we included the results of all these measures in the meta-analysis to form 15 comparisons (Figure 3). The meta-analysis showed no statistically significant difference (*P*=.29) in executive function between serious games and control groups (SMD –0.19, 95% CI –0.54 to 0.16). The statistical heterogeneity of the evidence was considerable (*P*<.001, I^2^=81%). The quality of the evidence was very low, as it was downgraded by 6 levels due to high risk of bias, heterogeneity, and imprecision (Multimedia Appendix 5).

The SMD of 2 comparisons seemed to be outliers (–2.15 [36] and 0.81 [31]), although characteristics of the studies in these comparisons were comparable to the other studies in this meta-analysis. Thus, we conducted a sensitivity analysis to check whether removing these outliers influenced the overall effect size and heterogeneity level. The sensitivity analysis showed that the difference in executive function between the groups remained insignificant (*P*=.17), but the heterogeneity substantially decreased from 81% to 31%.

**Figure 4:**
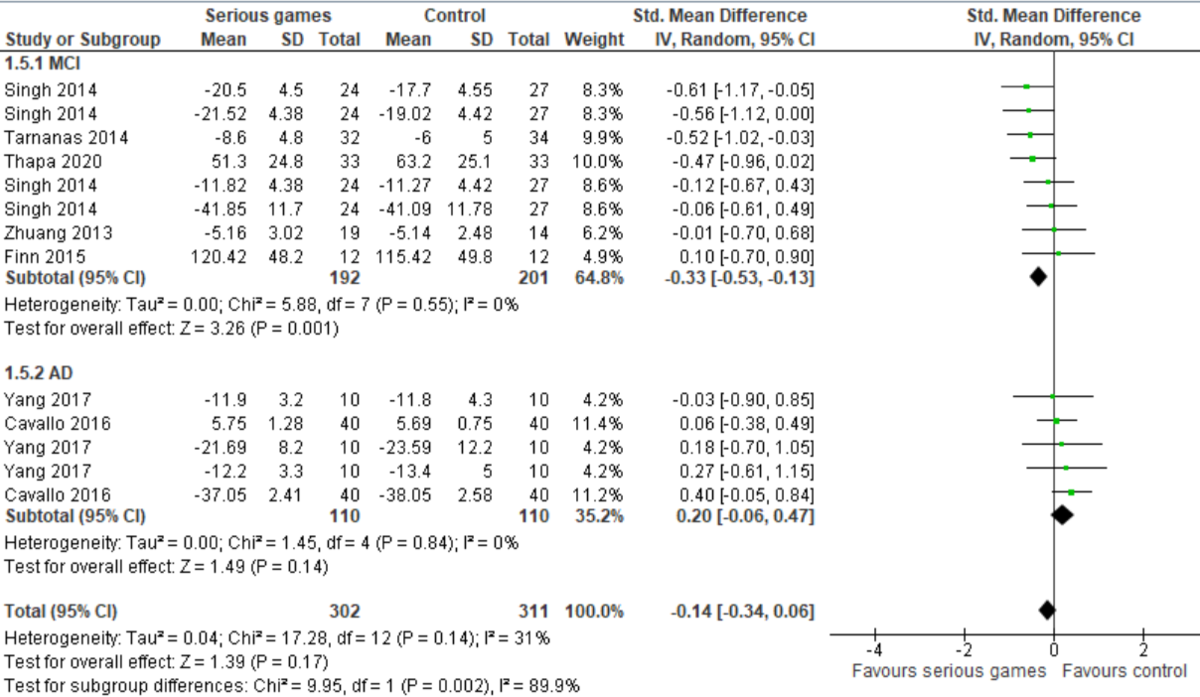
Forest plot of 7 studies (13 comparisons) comparing the effect of serious games on older adults with mild cognitive impairment (MCI) to their effect on older adults with Alzheimer disease (AD).

We conducted a subgroup analysis to assess whether the effect of serious games is based on the health conditions of participants. As shown in Figure 4, there was a statistically significant difference (*P*=.002) between the effect of serious games on executive functions among older adults with MCI (SMD 0.33) and their effect on executive functions among older adults with dementia (SMD 0.20) when compared with a control.

#### Serious Games Versus Conventional Exercises

**Figure 5:**
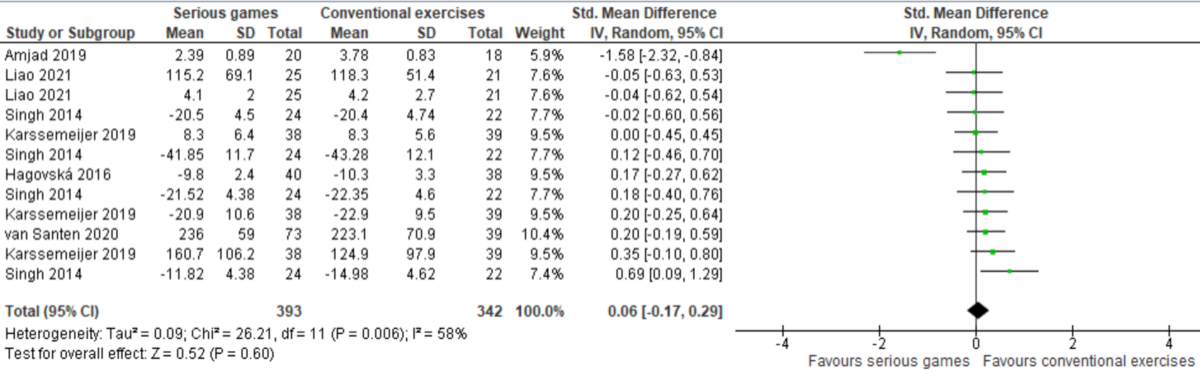
Forest plot of 6 studies (12 comparisons) comparing the effect of serious games to conventional exercises on executive functions.

In 6 studies [37-42], the effect of serious games was compared with conventional exercises. Of these studies, 3 evaluated executive function using more than one measure [37,41,42]. Therefore, we included the results of all these measures in the meta-analysis to form 12 comparisons (Figure 5). The meta-analysis showed no statistically significant difference (*P*=.60) in executive function between the serious games group and conventional exercises group (SMD 0.06, 95% CI –0.17 to 0.29). The statistical heterogeneity of the evidence was moderate (*P*=.006, I^2^=58%). The quality of the evidence was very low, as it was downgraded by 5 levels due to high risk of bias, heterogeneity, and imprecision (Multimedia Appendix 5).

The effect size in 1 study [38] seemed to be an outlier (–1.58). This could be attributed to the following reasons: (1) The sample size in this study was the smallest (n=44) in all the meta-analyzed studies, (2) it was the only study that used a videogame console (Xbox) as a platform for the serious game, and (3) the interventions in this study were delivered for a short period (6 weeks) in comparison with other studies included in this meta-analysis. Accordingly, we ran a sensitivity analysis to check whether removing this outlier influenced the overall effect size and heterogeneity level. The sensitivity analysis showed a statistically significant difference in executive functions (*P*=.03) between the groups, favoring conventional exercises over serious games (SMD 0.17, 95% CI 0.02 to 0.32). This difference was also clinically important, as the overall effect was outside the MCID boundaries (–0.085 to 0.085) and its CI did not cross the “no effect” line (zero effect). For this outcome, MCID boundaries were calculated as ±0.5 times the SMD value (0.17). The statistical heterogeneity of the evidence was not a concern (*P*=.85, I^2^=0%). The quality of this evidence was very low, as it was downgraded by 3 levels due to high risk of bias and imprecision.

**Figure 6:**
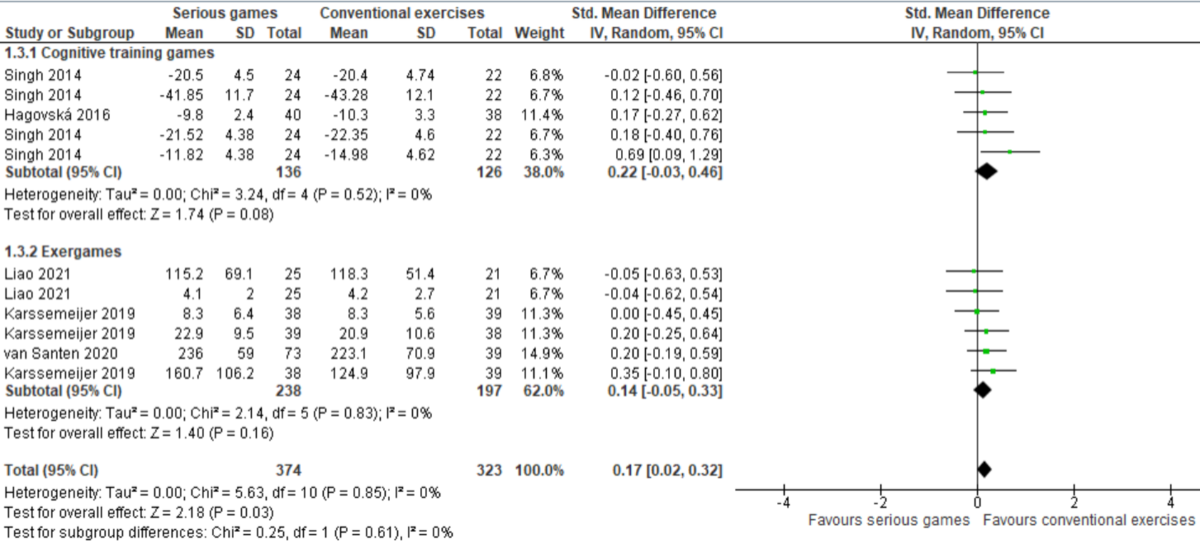
Forest plot of 5 studies (11 comparisons) comparing the effect of cognitive training games and exergames to conventional exercises.

In this comparison (ie, serious games vs conventional exercises), 2 types of serious games were used: cognitive training games and exergames. We conducted a subgroup analysis to investigate whether different types of serious games (ie, cognitive training games and exergames) have a different effect on executive functions (Figure 6). The subgroup analysis showed no statistically significant difference (*P*=.61) between cognitive training games (SMD 0.22) and exergames (SMD 0.14) in their effect on executive functions when compared with conventional exercises.

**Figure 7:**
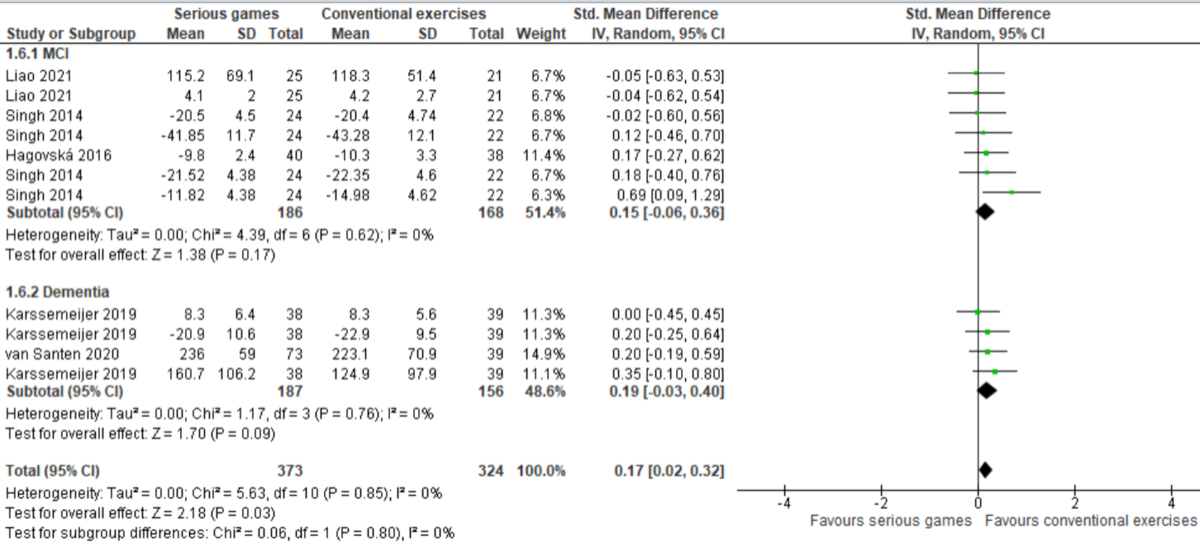
Forest plot of 5 studies (11 comparisons) comparing the effect of serious games on older adults with mild cognitive impairment (MCI) to their effect on older adults with dementia.

Further, we conducted a subgroup analysis to assess whether the effect of serious games is based on the health conditions of participants. As shown in Figure 7, there was no statistically significant difference (*P*=.80) between the effect of serious games on executive functions among older adults with MCI (SMD 0.15) and their effect on executive functions among older adults with dementia (SMD 0.19) when compared with conventional exercises.

#### Serious Games Versus Other Serious Games

The effect of serious games on executive function in comparison with other serious games was assessed in 4 studies [43-46]. Specifically, the first study compared the effect of a cognitive training game with exergames [45]. The study showed no statistically significant difference (*P*=.52) in executive functions between the groups [45]. The second study compared the effect of a cognitive training game that targets only memory and attention (COMCOG) with another cognitive training game that targets many cognitive abilities (ie, orientation, attention, memory, language, executive function, visuospatial function, calculation, and motor functions; Bettercog) [46]. This study found no statistically significant difference (*P*=.07) in executive functions between the groups [46].

**Figure 8:**
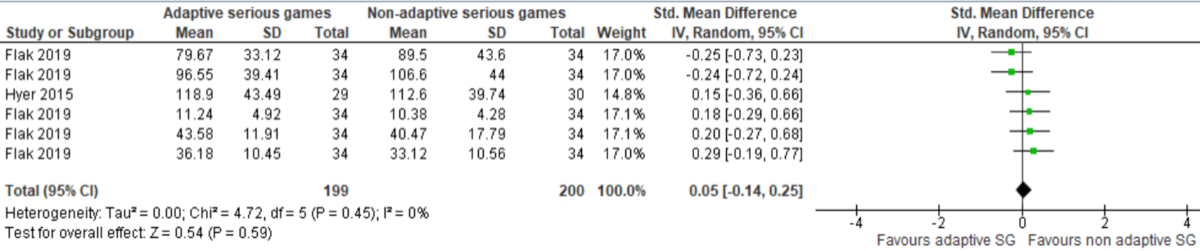
Forest plot of 2 studies (5 comparisons) comparing the effect of adaptive serious games to nonadaptive serious games.

The 2 remaining studies compared the effect of cognitive training games that adjust the level of difficulty of the tasks based on the individual’s mastery in each level (ie, adaptive games) with the same games but without adjustment of the level of difficulty of the tasks (ie, nonadaptive game) [43,44]. One of these studies assessed executive function using 4 different measures [43]. Thus, we ran a meta-analysis using these measures to form 5 comparisons. As shown in Figure 8, there was no statistically significant difference (*P*=.59) in executive functions between groups (SMD 0.05, 95% CI –0.14 to 0.25). The statistical heterogeneity of the evidence was not a concern (*P*=.45, I^2^=0%). The quality of the evidence was very low, as it was downgraded by 3 levels due to high risk of bias and imprecision (Multimedia Appendix 5).

## Discussion

### Principal Findings

This study summarized the evidence about the effectiveness of serious games for improving executive functions among older adults with cognitive impairment. Our meta-analyses showed that serious games are as effective as no or passive interventions at improving executive functions. Surprisingly, we found that conventional exercises are more effective than serious games at improving executive functions. However, our subgroup analysis showed that cognitive training games and exergames have a comparable effect on executive functions and they are as effective as conventional exercises at improving executive functions. We also found no difference between adaptive serious games and nonadaptive serious games at improving executive functions.

The lack of superiority of serious games over no or passive interventions and conventional exercises may be attributed to the following reasons: (1) The content of serious games in the meta-analyzed studies did not specifically target executive functions; (2) the target population (older adults with cognitive impairment) are less likely to be able to effectively play serious games; (3) most included studies assessed overall executive function rather than specific executive functions (eg, inhibition, impulse control, self-monitoring, task initiation, emotional control, flexible thinking), leaving it unclear whether the effect of serious games is different for different executive functions; and (4) the sample size in most included studies was small (≤100).

The findings of our review and those of previous reviews were consistent for some comparisons and different for others. Specifically, Lampit et al [20] summarized the evidence about the effect of cognitive training games on executive functions among healthy older adults in comparison with passive and active interventions. The review found cognitive training games are as effective as active interventions in improving executive functions; however, it showed that serious games are more effective at improving executive functions in comparison with no or passive interventions [20]. Another review compared the effect of cognitive training games on executive functions with any other interventions (passive or active interventions) [11]. The review demonstrated that cognitive training games are more effective than other interventions at improving executive functions among healthy older adults [11]. Our findings are inconsistent with the findings of these reviews [11,20]. This difference may be attributed to the fact that both reviews focused on healthy older adults only while the current review focused on older adults with cognitive impairment.

Meta-analyses from 2 other reviews showed that the effect of cognitive training games on executive functions among older adults with cognitive impairment is not statistically significant in comparison with other passive and active interventions [22,24]. Our findings are in line with the results of these reviews [22,24]. However, the main differences between the current review and these reviews are as follows: (1) The previous reviews focused only on a specific type of serious games (ie, cognitive training games), while the current review focused on all types of serious games; (2) they did not compare the effect of cognitive training games with a specific type of comparator (no or passive interventions, conventional exercise, other serious games); and (3) they included pilot RCTs and quasiexperiments, whereas the current review excluded such studies.

A systematic review conducted by Yen and Chiu [21] showed that exergames do not significantly improve executive functions among older adults in comparison with other passive and active interventions. Our findings are consistent with the finding of the previous review [21]. In contrast, another review found that exergames are more effective than passive and active interventions in improving executive functions among healthy older adults [19]. This contradictory finding may be attributed to 2 reasons: (1) Although the former review [21] focused on older adults with and without cognitive impairment, the latter review [19] focused on the older adults without cognitive impairment, and (2) the former review [21] assessed the effect of virtual reality exergames, while the latter review [19] examined the effect of exergames in general.

None of the previous reviews compared the effect of adaptive serious games with nonadaptive serious games on executive functions. However, a review compared the effect of adaptive serious games with that of nonadaptive serious games on working memory among older adults with cognitive impairment [47]. The review found no statistically significant difference in working memory between groups [47], and this is in line with our findings.

### Strengths and Limitations

#### Strengths

In comparison with previous reviews [11,18-24], this review is the first of its kind, to the best of our knowledge, that compares both the effect of serious games and their types on executive functions with a specific comparator (ie, no intervention, conventional exercises, and other serious games). Further, this review is the first of its kind to use the GRADE approach to appraise the quality of the evidence resulting from the meta-analyses, and this enables the reader to draw more accurate conclusions.

This review followed highly recommended guidelines for reporting systematic reviews (ie, PRISMA); thus, it can be considered a transparent and reproducible review. Our findings are based on RCTs, which are the most rigorous research method in studying cause-effect relationships [48]. Hence, the findings of this review are more likely to be reliable than findings generated from reviews that included other study designs such as pilot RCTs and quasiexperiments.

The risk of publication bias in this current review is not a concern, as the authors sought to retrieve as many relevant studies as possible through searching the most popular databases in information technology and health fields and grey literature databases, conducting backward and forward reference list checking, and using a well-developed search query. In addition, the risk of selection bias in this review is minimal because the study selection, data extraction, risk of bias assessment, and quality of evidence appraisal were conducted by 2 reviewers independently.

#### Limitations

The current review focused on the effectiveness of digital serious games in improving executive functions among older adults with cognitive impairment. For this reason, this review cannot comment on the effectiveness (1) of nondigital serious games or those used for nontherapeutic purposes (eg, screening or diagnosis), (2) at improving a specific executive function or other cognitive abilities (eg, attention, processing speed, memory), and (3) among other age groups or those without cognitive impairment.

In this review, the effect size for each study was estimated using postintervention data rather than the pre-post intervention change for each group; thereby, it is likely that the effect size is overestimated or underestimated. Postintervention outcome data were used because most studies did not report the mean and standard deviation for pre-post intervention change in executive functions for each group and the difference in executive functions between groups at baseline was not statistically significant in all studies.

This review assessed only the short-term effect of serious games by pooling only postintervention data rather than follow-up data, as the follow-up period was not consistent between the 5 studies that reported follow-up data. Thus, we cannot comment on the long-term effect of serious games on executive functions. It is likely that this review missed some relevant studies given that we excluded studies that were published before 2010, written in a language other than English, quasiexperiments, and pilot RCTs.

### Practical and Research Implications

#### Practical Implications

This review showed no superior effect of serious games compared with no or passive interventions and conventional exercises on executive functions among older adults with cognitive impairment. Further, there was no difference between adaptive serious games and nonadaptive serious games at improving executive functions among older adults with cognitive impairment. However, readers should cautiously interpret our findings for the following reasons: (1) The quality of evidence ranged between very low to low due mainly to high risk of bias, high heterogeneity, and imprecision of the estimated total effect sizes; (2) the number of studies included in several meta-analyses was small; and (3) the sample size in most studies included in the meta-analyses was small. Accordingly, serious games should not be offered or used for improving executive functions among older adults with cognitive functions until more robust evidence is available. This is a call to action for researchers, clinicians, and game developers to continue improving their work and focus on addressing the limitations and concerns discussed earlier.

Smart mobile devices (ie, tablets and smartphones) were not used in any study included in this review. Smart mobile devices are particularly appealing, as they are cheaper, more accessible, and more pervasive than computers and gaming consoles. Globally, the number of mobile devices and mobile users in 2021 were about 15 billion and 7.1 billion, respectively, and these figures are expected to rise considerably by 2025 [49]. There is an opportunity for smart device app developers as well as serious game developers to create and tailor serious games that target executive functions of older adults with cognitive impairment and can be played via mobile devices.

#### Research Implications

This review addressed the research gap related to the short-term effect of serious games on executive functions among older adults with cognitive impairment. However, further reviews are needed to address the following research gaps: (1) the long-term effect of serious games, (2) the effect of serious games on specific executive functions (eg, inhibition, impulse control, self-monitoring, flexible thinking) and on other cognitive abilities (eg, attention, processing speed, learning), and (3) the effect of serious games among people of different age groups with or without cognitive impairment.

Most included studies were conducted in developed countries; thereby, the generalizability of this review’s findings to developing countries may be limited given the varying nature of their cultures and socioeconomic conditions. Researchers should carry out more studies in developing countries. The mean and standard deviation for pre-post intervention change in executive functions for each group were not reported by most of the included studies. To calculate a more accurate effect size for each study, we urge researchers to report such information in their future publications.

Previous reviews showed that the effect of exergames on executive functions among healthy older adults was investigated by many studies [19,21,23]. However, in the current review, only 3 studies examined the effect of exergames on executive functions among older adults with cognitive impairment. Further studies are required to bridge this research gap. In this review, serious games were compared with conventional cognitive training by only 1 study, and adaptive serious games were compared with nonadaptive serious games by only 2 studies. To draw more definitive conclusions, these comparisons should be examined by further trials.

Only 3 of the included studies were judged to have a low overall risk of bias, as the remaining studies had issues mainly in the randomization process or selection of the reported results (ie, unpublished protocol or analysis plan). To minimize the risk of bias, researchers should conduct and report their trials according to recommended guidelines or tools such as the RoB-2 [27].

### Conclusion

The evidence from this review showed no superior effect of serious games compared with no or passive interventions and conventional exercises on executive functions among older adults with cognitive impairment. However, this should not be considered a definitive conclusion for the following reasons: (1) The quality of evidence ranged between very low to low due mainly to high risk of bias, high heterogeneity, and imprecision of the estimated total effect sizes; (2) the number of studies included in several meta-analyses was small; and (3) the sample size in most studies included in the meta-analyses was small. Therefore, until more robust evidence is available, serious games should not be offered by health care providers nor used by patients for improving executive functions among older adults with cognitive impairment. Further reviews are needed to assess the long-term effect of serious games on specific executive functions or other cognitive abilities among people of different age groups with or without cognitive impairment. Additional RCTs should be conducted to examine the effect of exergames on executive functions among older adults with cognitive impairment and to compare the effect of serious games with conventional exercises.

## Appendix

Multimedia Appendix 1 PRISMA checklist.

Multimedia Appendix 2 Search strategy.

Multimedia Appendix 3 Data extraction form.

Multimedia Appendix 4 Reviewers’ judgments about each “risk of bias” domain for each included study.

Multimedia Appendix 5 GRADE Profile for comparison of serious games to control, conventional exercises, and non-adaptive serious games for executive functions.

## Abbreviations

AD: Alzheimer disease
CBT: cognitive behavioral therapy
DALYs: Disability-adjusted life years
MCI: mild cognitive impairment
MCID: minimal clinically important difference
MMSE: Mini-Mental State Examination
PRISMA: Preferred Reporting Items for Systematic Reviews and Meta-Analyses
RCT: Randomized Controlled Trial
RoB-2: Risk-of-Bias 2
SMD: standardized mean difference
WHO: World Health Organization
