# Supplementary material for: Effectiveness of Serious Games for Improving Executive Functions Among Older Adults With Cognitive Impairment: Systematic Review and Meta-analysis
Source: JMIR Serious Games. 2022 Jul 25;10(3):e36123. doi: 10.2196/36123 (PMC9361143; doi:10.2196/36123)
Supplement: Multimedia Appendix 2 [file games_v10i3e36123_app2.docx]

**Appendix 2 Search strategy**

Database(s): **Ovid MEDLINE(R) ALL**1946 to November 10, 2021
Search Strategy:

| **#** | **Searches** | **Results** |
| --- | --- | --- |
| 1 | exp Cognitive Dysfunction/ | 24076 |
| 2 | "cognitive impair*".tw. | 71871 |
| 3 | "cognitive disorder*".tw. | 4720 |
| 4 | "Cognitive Dysfunction".tw. | 15020 |
| 5 | exp Dementia/ | 178288 |
| 6 | dementia*.tw. | 118323 |
| 7 | exp Alzheimer Disease/ | 101415 |
| 8 | Alzheimer*.tw. | 157340 |
| 9 | exp Randomized Controlled Trials as Topic/ | 150550 |
| 10 | "randomized controlled trial*".tw. | 155130 |
| 11 | "randomised controlled trial*".tw. | 48143 |
| 12 | "randomized control trial*".tw. | 8122 |
| 13 | "randomised control trial*".tw. | 2052 |
| 14 | "clinical trial*".tw. | 406889 |
| 15 | experiment*.tw. | 2176503 |
| 16 | Video Games/ | 6178 |
| 17 | "serious gam*".tw. | 845 |
| 18 | "game-based".tw. | 756 |
| 19 | "videogame*".tw. | 803 |
| 20 | "video game*".tw. | 3663 |
| 21 | "virtual reality game*".tw. | 122 |
| 22 | "virtual reality-based game*".tw. | 3 |
| 23 | "Augmented Reality-based game*".tw. | 0 |
| 24 | "Augmented Reality game*".tw. | 35 |
| 25 | "gamification".tw. | 676 |
| 26 | exergame*.tw. | 563 |
| 27 | "Applied game*".tw. | 22 |
| 28 | 1 or 2 or 3 or 4 or 5 or 6 or 7 or 8 | 328986 |
| 29 | 9 or 10 or 11 or 12 or 13 or 14 or 15 | 2801717 |
| 30 | 16 or 17 or 18 or 19 or 20 or 21 or 22 or 23 or 24 or 25 or 26 or 27 | 10105 |
| 31 | 28 and 29 and 30 | 95 |
| 32 | limit 31 to (english language and yr="2010 -Current") | 89 |

Database(s): **Embase**1996 to 2021 Week 45
Search Strategy:

| **#** | **Searches** | **Results** |
| --- | --- | --- |
| 1 | exp Cognitive Dysfunction/ | 485195 |
| 2 | "cognitive impair*".tw. | 109403 |
| 3 | "cognitive disorder*".tw. | 7371 |
| 4 | "Cognitive Dysfunction".tw. | 22045 |
| 5 | exp Dementia/ | 349122 |
| 6 | dementia*.tw. | 156242 |
| 7 | exp Alzheimer Disease/ | 197082 |
| 8 | Alzheimer*.tw. | 199506 |
| 9 | exp Randomized Controlled Trials as Topic/ | 208464 |
| 10 | "randomized controlled trial*".tw. | 198960 |
| 11 | "randomised controlled trial*".tw. | 62892 |
| 12 | "randomized control trial*".tw. | 12545 |
| 13 | "randomised control trial*".tw. | 3403 |
| 14 | "clinical trial*".tw. | 545745 |
| 15 | experiment*.tw. | 1945910 |
| 16 | Video Games/ | 4368 |
| 17 | "serious gam*".tw. | 959 |
| 18 | "game-based".tw. | 833 |
| 19 | "videogame*".tw. | 955 |
| 20 | "video game*".tw. | 4423 |
| 21 | "virtual reality game*".tw. | 165 |
| 22 | "virtual reality-based game*".tw. | 4 |
| 23 | "Augmented Reality-based game*".tw. | 0 |
| 24 | "Augmented Reality game*".tw. | 29 |
| 25 | "gamification".tw. | 741 |
| 26 | exergame*.tw. | 567 |
| 27 | "Applied game*".tw. | 21 |
| 28 | 1 or 2 or 3 or 4 or 5 or 6 or 7 or 8 | 545976 |
| 29 | 9 or 10 or 11 or 12 or 13 or 14 or 15 | 2789320 |
| 30 | 16 or 17 or 18 or 19 or 20 or 21 or 22 or 23 or 24 or 25 or 26 or 27 | 9688 |
| 31 | 28 and 29 and 30 | 145 |
| 32 | limit 31 to (english language and yr="2010 -Current") | 139 |
| 33 | limit 32 to exclude medline journals | 36 |

Database(s): **APA PsycInfo**2002 to November Week 2 2021
Search Strategy:

| **#** | **Searches** | **Results** |
| --- | --- | --- |
| 1 | exp Cognitive Dysfunction/ | 38332 |
| 2 | "cognitive impair*".tw. | 37215 |
| 3 | "cognitive disorder*".tw. | 2565 |
| 4 | "Cognitive Dysfunction".tw. | 5999 |
| 5 | exp Dementia/ | 63790 |
| 6 | dementia*.tw. | 53275 |
| 7 | exp Alzheimer Disease/ | 38674 |
| 8 | Alzheimer*.tw. | 52253 |
| 9 | exp Randomized Controlled Trials as Topic/ | 0 |
| 10 | "randomized controlled trial*".tw. | 29289 |
| 11 | "randomised controlled trial*".tw. | 5915 |
| 12 | "randomized control trial*".tw. | 2114 |
| 13 | "randomised control trial*".tw. | 413 |
| 14 | "clinical trial*".tw. | 34023 |
| 15 | experiment*.tw. | 265822 |
| 16 | Video Games/ | 7605 |
| 17 | "serious gam*".tw. | 958 |
| 18 | "game-based".tw. | 1575 |
| 19 | "videogame*".tw. | 1001 |
| 20 | "video game*".tw. | 5380 |
| 21 | "virtual reality game*".tw. | 67 |
| 22 | "virtual reality-based game*".tw. | 1 |
| 23 | "Augmented Reality-based game*".tw. | 0 |
| 24 | "Augmented Reality game*".tw. | 51 |
| 25 | "gamification".tw. | 764 |
| 26 | exergame*.tw. | 321 |
| 27 | "Applied game*".tw. | 19 |
| 28 | 1 or 2 or 3 or 4 or 5 or 6 or 7 or 8 | 119231 |
| 29 | 9 or 10 or 11 or 12 or 13 or 14 or 15 | 327756 |
| 30 | 16 or 17 or 18 or 19 or 20 or 21 or 22 or 23 or 24 or 25 or 26 or 27 | 11646 |
| 31 | 28 and 29 and 30 | 51 |
| 32 | limit 31 to (english language and yr="2010 -Current") | 45 |

Database(s): **CINHAL (EBSCO)**

| **#** | **Query** | **Results** |
| --- | --- | --- |
| S1 | SU cognitive impairment OR TI "cognitive impair*" OR AB "cognitive impair*" | 25842 |
| S2 | SU cognitive dysfunction [mesh] OR TI "Cognitive Dysfunction" OR AB "Cognitive Dysfunction" | 3596 |
| S3 | SU cognitive disorder* OR TI "cognitive disorder*" OR AB "cognitive disorder* | 4716 |
| S4 | SU Dementia OR TI Dementia OR AB Dementia | 68407 |
| S5 | SU alzheimer's disease OR TI alzheimer's disease OR AB alzheimer's disease | 43566 |
| S6 | SU Randomized Controlled Trials OR TI "Randomized Controlled Trial*" OR AB "Randomized Controlled Trial" | 148101 |
| S7 | TI "Randomised Controlled Trial*" OR AB "Randomised Controlled Trial*" | 25432 |
| S8 | TI "Randomized Control Trial*" OR AB "Randomized Control Trial*" | 3896 |
| S9 | TI "Randomised Control Trial*" OR AB "Randomised Control Trial*" | 1143 |
| S10 | TI "clinical trial*" OR AB "clinical trial*" | 119088 |
| S11 | TI experiment* OR AB experiment* | 146276 |
| S12 | SU Video Games OR TI "Video Game*" OR "Video Game*" | 5815 |
| S13 | SU serious games OR TI "serious gam*" OR AB "serious gam*" | 425 |
| S14 | TI "game-based" OR AB "game-based" | 459 |
| S15 | TI "videogame*" OR AB "videogame*" | 320 |
| S16 | TI "virtual reality game*" OR AB "virtual reality game*" | 71 |
| S17 | TI "Augmented Reality-based game*" OR AB "Augmented Reality-based game*" | 0 |
| S18 | TI "Augmented Reality game*" OR AB "Augmented Reality game*" | 22 |
| S19 | TI "gamification" OR AB "gamification" | 402 |
| S20 | TI exergam* OR AB exergam* | 398 |
| S21 | TI "Applied game*" OR AB "Applied game*" | 10 |
| S22 | S1 OR S2 OR S3 OR S4 OR S5 | 115717 |
| S23 | (S6 OR S7 OR S8 OR S9 OR S10 OR S11) | 393374 |
| S24 | S12 OR S13 OR S14 OR S15 OR S16 OR S17 OR S18 OR S19 OR S20 OR S21 | 6945 |
| S25 | (S12 OR S13 OR S14 OR S15 OR S16 OR S17 OR S18 OR S19 OR S20 OR S21) AND (S22 AND S23 AND S24) | 37 |
| S26 | Limiters - English Language (S25) | 32 |

| **Database** | **Query** | **Results** |
| --- | --- | --- |
| **Scopus** | ( TITLE-ABS-KEY ( "serious gam*" OR "game-based" OR "videogame*" OR "video game*" OR "virtual reality game*" OR "virtual reality-based game*" OR "Augmented Reality-based game*" OR "Augmented Reality game*" OR "gamification" OR gamified OR exergam* OR "Applied game*" ) AND TITLE-ABS-KEY ( "cognitive impair*" OR "cognitive disorder*" OR "Cognitive Dysfunction" OR dementia* OR alzheimer* ) AND TITLE-ABS-KEY ( "randomized controlled trial*" OR "randomised controlled trial*" OR "randomized control trial*" OR "randomised control trial*" OR "clinical trial*" OR experiment*  ) ) AND ( LIMIT-TO ( PUBYEAR , 2021 ) OR LIMIT-TO ( PUBYEAR , 2020 ) OR LIMIT-TO ( PUBYEAR , 2019 ) OR LIMIT-TO ( PUBYEAR , 2018 ) OR LIMIT-TO ( PUBYEAR , 2017 ) OR LIMIT-TO ( PUBYEAR , 2016 ) OR LIMIT-TO ( PUBYEAR , 2015 ) OR LIMIT-TO ( PUBYEAR , 2014 ) OR LIMIT-TO ( PUBYEAR , 2013 ) OR LIMIT-TO ( PUBYEAR , 2012 ) OR LIMIT-TO ( PUBYEAR , 2011 ) OR LIMIT-TO ( PUBYEAR , 2010 ) ) AND ( LIMIT-TO ( LANGUAGE , "English" ) ) AND ( LIMIT-TO ( DOCTYPE , "ar" ) OR LIMIT-TO ( DOCTYPE , "cp" ) ) | 176 |
| **IEEE Xplore** | ("Abstract":"serious game" OR "Abstract":"serious games" OR "Abstract":"game-based" OR "Abstract":"videogames" OR "Abstract":"video games" OR "Abstract":"videogame" OR "Abstract":"video game" OR "Abstract":"virtual reality game" OR "Abstract":"virtual reality games" OR "Abstract":"Augmented Reality game" OR "Abstract":"Augmented Reality games" OR "Abstract":"gamification" OR "Abstract":gamified OR "Abstract":exergam* OR "Abstract":"Applied game") AND ("Abstract":"cognitive impair*" OR "Abstract": "cognitive disorder*" OR "Abstract": "Cognitive Dysfunction" OR "Abstract": dementia OR "Abstract": alzheimer*) | 63 |
| **ACM Digital library** | *[[Abstract: "cognitive impair*"] OR [Abstract: "cognitive disorder*"] OR [Abstract: "cognitive dysfunction"] OR [Abstract: dementia*] OR [Abstract: alzheimer*]] AND [[Abstract: "serious gam*"] OR [Abstract: "game-based"] OR [Abstract: "videogame*"] OR [Abstract: "video game*"] OR [Abstract: "virtual reality game*"] OR [Abstract: "virtual reality-based game*"] OR [Abstract: "augmented reality-based game*"] OR [Abstract: "augmented reality game*"] OR [Abstract: "gamification"] OR [Abstract: gamified] OR [Abstract: exergam*] OR [Abstract: "applied game*"]] AND [[All: "randomized controlled trial*"] OR [All: "randomised controlled trial*"] OR [All: "randomized control trial*"] OR [All: "randomised control trial*"] OR [All: "clinical trial*"] OR [All: experiment*]] AND [Publication Date: (01/01/2010 TO 12/31/2021)]* | 7 |
| **Google Scholar** | ("cognitive impair*" OR "cognitive disorder*" OR "Cognitive Dysfunction" OR dementia* OR alzheimer*) AND ("serious gam*" OR "game-based" OR exergam*) AND ("controlled trial*" OR "control trial*") | 100 |
