# Supplementary material for: Effectiveness of Serious Games for Improving Executive Functions Among Older Adults With Cognitive Impairment: Systematic Review and Meta-analysis
Source: JMIR Serious Games. 2022 Jul 25;10(3):e36123. doi: 10.2196/36123 (PMC9361143; doi:10.2196/36123)
Supplement: Multimedia Appendix 5 [file games_v10i3e36123_app5.doc]

| **Appendix 5 GRADE Profile for comparison of serious games to control, conventional exercises, and non-adaptive serious games for executive functions** | | | | | | | | | | | |
| --- | --- | --- | --- | --- | --- | --- | --- | --- | --- | --- | --- |
| **Certainty assessment** | | | | | | | **Summary of findings** | | | | |
| **Participants (studies) Follow-up** | **Risk of bias** | **Inconsistency** | **Indirectness** | **Imprecision** | **Publication bias** | **Overall certainty of evidence** | **Study event rates (%)** | | **Relative effect (95% CI)** | **Anticipated absolute effects** | |
|  |  |  |  |  |  |  | **Control** | **Serious games** |  | **Risk with** | **Risk difference with Serious games** |
| **Serious games vs. Control** | | | | | | | | | | | |
| 759 (7 RCTs)  (15 comparisons) | very serious^a^ | very serious^b^ | not serious | very serious^c,d^ | none | ⨁◯◯◯ Very low | 385 | 374 | - | - | SMD **0.19 lower** (0.54 lower to 0.16 higher) |
| **Serious games vs. Conventional exercises** | | | | | | | | | | | |
| 735 (6 RCTs)  (12 comparisons) | very serious^e^ | serious^f^ | not serious | very serious^c,g^ | none | ⨁◯◯◯ Very low | 342 | 393 | - | - | SMD **0.06 higher** (0.17 lower to 0.29 higher) |
| **Serious games vs. Conventional exercises - Cognitive training games** | | | | | | | | | | | |
| 262 (2 RCTs)  (5 comparisons) | serious^h^ | not serious | not serious | serious^i,j^ | none | ⨁⨁◯◯ Low | 126 | 136 | - | - | SMD **0.22 lower** (0.03 lower to 0.46 higher) |
| **Serious games vs. Conventional exercises - Exergames** | | | | | | | | | | | |
| 435 (3 RCTs)  (6 comparisons) | very serious^k^ | not serious | not serious | serious ^j,l^ | none | ⨁◯◯◯ Very low | 197 | 238 | - | - | SMD **0.14 higher** (0.05 lower to 0.33 higher) |
| **Adaptive serious games vs. Non-adaptive serious games** | | | | | | | | | | | |
| 399 (2 RCTs)  (5 comparisons) | serious^h^ | not serious | not serious | very serious^c,m^ | none | ⨁⨁◯◯ Low | 200 | 199 | - | - | SMD **0.05 higher** (0.14 lower to 0.25 higher) |

**CI:** confidence interval; **SMD:** standardised mean difference

#### Explanations

a. Evidence was downgraded by 2 levels because only 4 of 15 meta-analyzed studies were judged to have a low risk of bias, this is due to issues mainly in the randomization process and selection of the reported results in the remaining studies.

b. Evidence was downgraded by 2 levels as P<0.001 and I square=81%, indicating high heterogeneity.

c. Evidence was downgraded by 2 levels because 95% CI crosses the two MID boundaries for this outcome.

d. MID for this outcome, calculated as ± 0.5 times the standardized mean difference (SMD), is ± 0.095

e. Evidence was downgraded by 2 levels because there were some concerns in half of the meta-analyzed studies due to issues mainly in the randomization process and selection of the reported results.

f. Evidence was downgraded by 1 level as P<0.001 and I square=58%, indicating moderate heterogeneity.

g. MID for this outcome, calculated as ± 0.5 times the standardized mean difference (SMD), is ± 0.03

h. Evidence was downgraded by 1 level because there were some concerns in one study due to issues mainly in the selection of the reported results.

i. MID for this outcome, calculated as ± 0.5 times the standardized mean difference (SMD), is ±0.11

j. Evidence was downgraded by 1 level because 95% CI crosses one of MID boundaries for this outcome.

k. Evidence was downgraded by 2 levels because none of the meta-analyzed studies were judged to have a low risk of bias, this is due to issues mainly in the randomization process and missing outcome data

l. MID for this outcome, calculated as ± 0.5 times the standardized mean difference (SMD), is ± 0.07

m. MID for this outcome, calculated as ± 0.5 times the standardized mean difference (SMD), is ± 0.025
